# Canadian Rhinology Working Group consensus statement: biologic therapies for chronic rhinosinusitis

**DOI:** 10.1186/s40463-021-00493-2

**Published:** 2021-03-09

**Authors:** Andrew Thamboo, S. Kilty, I. Witterick, Y. Chan, C. J. Chin, A. Janjua, A. Javer, J. Lee, E. Monterio, B. Rotenberg, J. Scott, K. Smith, D. D. Sommer, L. Sowerby, M. Tewfik, E. Wright, M. Desrosiers

**Affiliations:** 1 Division of Otolaryngology-Head and Neck Surgery, Department of Surgery, University of British Columbia, 2600-1081 Burrard Street, Vancouver, British Columbia V6Z 1Y6 Canada; 2 Department of Otolaryngology-Head and Neck Surgery, The University of Ottawa and The Ottawa Hospital, Ottawa, ON Canada; 3 Department of Otolaryngology-Head & Neck Surgery, University of Toronto, Toronto, ON Canada; 4 Division of Otolaryngology-Head and Neck Surgery, Department of Surgery, Dalhousie University, Halifax, NS Canada; 5 Department of Otolaryngology-Head and Neck Surgery, Western University, London, ON Canada; 6 Department of Otolaryngology-Head and Neck Surgery, University of Manitoba, Winnipeg, MB Canada; 7 Division of Otolaryngology-Head and Neck Surgery, Department of Surgery, McMaster University, Hamilton, ON Canada; 8 Department of Otolaryngology-Head and Neck Surgery, McGill University, Montreal, QC Canada; 9 Division of Otolaryngology-Head and Neck Surgery, Department of Surgery, University of Alberta, Edmonton, AB Canada; 10 Division of Otolaryngology-Head and Neck Surgery, Centre Hospitalier de l’University de Montreal, Montreal, QC Canada

**Keywords:** Chronic Rhinosinusitis, Biologics, Chronic rhinosinusitis with nasal polyposis, Type 2 inflammation

## Abstract

**Background:**

Recent evidence suggests that biologic therapy with targeted activity within the Type 2 inflammatory pathway can improve the clinical signs and symptoms of chronic rhinosinusitis with nasal polyposis (CRSwNP). There remains a population in CRSwNP that despite medical therapy and endoscopic sinus surgery have persistent signs and symptoms of disease. Therefore, biologics, monoclonal antibody agents, could be beneficial therapeutic treatments for these patients. There have been eight randomized, double-blind, placebo-controlled trails performed for CRSwNP targeted components of the Type 2 inflammatory pathway, notably interleukin (IL)-4, IL-5 and IL-13, IL-5R, IL-33, and immunoglobulin (Ig)E. However, there are no formal recommendations for the optimal use of biologics in managing Chronic Rhinosinusitis (CRS) within the Canadian health care environment.

**Methods:**

A Delphi Method process was utilized involving three rounds of questionnaires in which the first two were completed individually online and the third was discussed on a virtual platform with all the panelists. 17 fellowship trained rhinologists across Canada evaluated the 28 original statements on a scale of 1–10 and provided comments. A rating within 1–3 indicated disagreement, 8–10 demonstrated agreement and 4–7 represented being neutral towards a statement. All ratings were quantitively reviewed by mean, median, mode, range and standard deviation. Consensus was defined by removing the highest and lowest of the scores and using the “3 point relaxed system”.

**Results:**

After three rounds, a total of 11 statements achieved consensus. This white paper only contains the final agreed upon statements and clear rationale and support for the statements regarding the use of biologics in patients with CRS.

**Conclusion:**

This white paper provides guidance to Canadian physicians on the use of biologic therapy for the management of patients with CRS, but the medical and surgical regimen should ultimately be individualized to the patient. As more biologics become available and additional trials are published we will provide updated versions of this white paper every few years.

**Graphical abstract:**

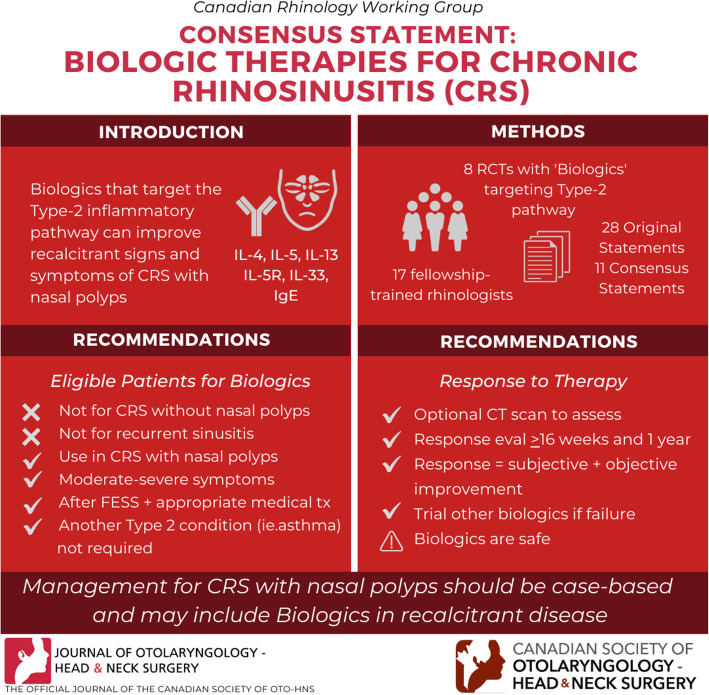

## Background

The Canadian clinical practice guideline for the management of chronic rhinosinusitis (CRS) provides the framework for the medical and surgical management of patients with CRS [1]. Recent developments in the medical management of CRS have occurred due to an improved understanding of the disease pathophysiology, including Type 2 inflammation, which has led to therapy that is able to modulate the immune response present in CRS. Despite improvements in delivery methods (budesonide irrigation [2], Xhance delivery device (Optinose®, Yardley, PA.) [3], etc.), compliance strategies, and refinements in surgical technique, there continues to be a CRS patient population that has a limited response to currently available strategies. Recent clinical trial data has suggested that biologic therapy with targeted activity within the Type 2 inflammatory pathway can improve the clinical signs and symptoms of chronic rhinosinusitis with nasal polyposis (CRSwNP) in patients with medically and/or surgically recalcitrant disease. This has given rise to an interest in biologic monoclonal antibody agents as therapeutic treatments for CRS. However, until now, high-quality evidence for the use of biologic therapies in managing CRS has not been available and thus no formal recommendations exist for their optimal use within the Canadian health care environment. This white paper aims to fill this knowledge gap by offering guidance to Canadian physicians on the use of biologic therapy for the management of patients with CRS as of September 2020.

In the current environment, patients with CRSwNP whose disease is poorly responsive to medical therapy, with persistent signs and symptoms of disease, may undergo endoscopic sinus surgery (ESS). In general, the objective of this surgery is not curative, but rather to decrease the inflammatory load, thereby increasing nasal airflow and sinus drainage, and to improve access for facilitation of topical medication delivery to the paranasal sinuses. Although this approach is successful in the majority of patients, there remains a population in whom lasting disease control remains elusive. Despite advances in surgical techniques and expertise, and combined with preventative postoperative treatments, recurrence of disease may be seen in some patients within six months. Furthermore, within five years, up to 10–30% of patients with nasal polyps require a revision surgery [4–6]. After surgery, patients rely on off-label use of saline and steroid combination irrigations, topical intranasal corticosteroid sprays, oral corticosteroids and antibiotics for control of symptoms which may be followed by additional surgery if symptoms persist. While beneficial in most cases, in patient groups with more severe disease, these medical therapies may offer control for only a short period of time, and thus the quality of life continues to be considerably impaired. In the face of persistent paranasal sinus disease, management of co-morbid asthma may be more difficult, leading to increased use of oral corticosteroids for polyp or asthma control [7]. Repeated and prolonged use of short-courses of oral prednisone have additive systemic adverse effects [8], and repeated courses of antibiotics may promote the development of bacterial resistance. Oral antibiotics are not routinely considered for CRSwNP but can be provided when patients experience purulence.

### Rationale for use of biologics in CRS

Biologic agents, or biologics, are created to target specific immune cells or mediators in an inflammatory cascade that are responsible for the progression of a disease [9]. In CRS, agents currently approved or under assessment for CRSwNP target components of the Type 2 inflammatory pathway, notably interleukin (IL)-4, IL-5 and IL-13, IL-5R, IL-33, and immunoglobulin (Ig)E [9]. All studies that have been conducted to date have been trialed in CRSwNP populations, except for one study that also included chronic rhinosinusitis without nasal polyposis (CRSsNP) patients [10]. As of September 2020, there have been eight randomized, double-blind, placebo-controlled trails performed using biologics that target the previously mentioned inflammatory mediators and one trial still underway that targets IL-33. The details of the eight completed trials are summarized in Table 1.

**Table 1:** Results of biologic trials for CRSwNP

Study	Treatment	Study sample size	Diagnosis	Comorbidities	Study Outcomes	Results
Nasal IL5 levels determine the response to anti-IL-5 treatment in patients with nasal polyps. Gevaert et al. (2006) [11]	Reslizumab (anti-IL-5)	*n* = 24 (Placebo *n* = 8 / 1 mg/kg Treatment *n* = 8 / 3 mg/kg Treatment *n* = 8)	CRSwNP	Asthma: *n* = 18	Nasal polyp score, adverse events, NPIF, disease symptoms score, blood and serum markers	- Total nasal polyp score was only significantly decreased in the 1 mg/kg group at week 12 - No significant difference in NPIF or disease symptoms score at any time point in treatment groups compared to placebo. - Significant decrease in blood eosinophil counts groups sustained until week 8 and Serum ECP and Secreted IL-5Rα until week 4 in both treatment groups
A randomized, double-blind, placebo-controlled trial of anti-IgE for chronic rhinosinusitis. Pinto et al. (2010) [12]	Omalizumab (anti-IgE)	*N* = 14 (Placebo *n* = 7 / Treatment *n* = 7)	CRSwNP/ Crisp	Asthma: All patients	Snot-22, SF-36, nasal polyp size, CT scan opacification percentage, adverse events, NPIF, eosinophil count, UPSIT	No significant differences in polyp size, CT scan opacification percentage, SNOT-22 score, NPIF in the omalizumab group compared to placebo Improvement in UPSIT smell test score but not statistically significant and no significant differences in SF-36 except for the one domain, Vitality, between omalizumab and placebo group
Mepolizumab, a humanized anti-IL-5 mAb, as a treatment option for severe nasal polyposis. Gevaert et al. (2011) [13]	Mepolizumab (anti-IL-5)	*n* = 30 (Placebo *n* = 10 / Treatment *n* = 20)	CRSwNP refractory to corticosteroid therapy	Asthma: *n* = 23 Allergies: *n* = 14 Aspirin intolerance: *n* = 5	Disease symptom scores, Adverse events, nasal polyp score, CT scan score, NPIF, blood and serum markers	- Significant improvement in total polyp score and CT scan scores from baseline in the mepolizumab group compared to placebo - No significant difference in disease symptoms scores or NPIF - Significant reduction of blood eosinophil counts and serum ECP and serum IL-5Rα levels at week 8 in mepolizumab group - Nasal IL-5Rα, IL-6, IL-1β, and MPO levels were significantly reduced in the mepolizumab group
Omalizumab is effective in allergic and nonallergic patients with nasal polyps and asthma. Gevaert et al. (2013) [14]	Omalizumab (anti-IgE)	*n* = 24 (Placebo *n* = 8 / Treatment *n* = 15)	CRSwNP with asthma	Asthma: All patients Allergies: *n* = 13 Aspirin intolerance: *n* = 12	Disease symptom scores, adverse events, RSOM-31, AQLQ, SF-36, polyp size and total overall polyp score, LMK Score, FEV_1_ and PEF, and blood and serum markers	- Significant reduction in polyp size, improvement in LMK scores in Omalizumab group after 16 weeks Significant decrease in symptom scores for Omalizumab group: nasal congestion, anterior rhinorrhea, loss of sense of smell, dyspnea Significant improvement in SF-36 of physical health, RSOM-31 of sleep and general symptoms and AQLQ after Omalizumab treatment No significant changes in blood and serum markers
Effect of Subcutaneous Dupilumab on Nasal Polyp Burden in Patients With Chronic Sinusitis and Nasal Polyposis: A Randomized Clinical Trial. Bachert et al. (2016) [15]	Dupilumab (anti-IL-4/IL-13)	*n* = 60 (Placebo *n* = 30 / Treatment *n* = 30)	CRSwNP refractory to intranasal corticosteroid therapy	Asthma: *n* = 35 Allergies: *n* = 38 Aspirin intolerance: *n* = 12	SNOT-22, VAS, adverse events, Endoscopic polyp score, LMK Score, UPSIT, NPIF, FEV_1_, ACQ-5, patient-rated disease severity symptoms	- Statistically significant improvement in SNOT-22 and UPSIT in the dupilumab group vs placebo - Statistically significant difference of least squares mean change in bilateral endoscopic nasal polyp score and LMK CT total scores between the treatment and placebo group - Statistically significant reduction of IgE, and plasma eotaxin-3 with dupilumab vs placebo
Reduced need for surgery in severe nasal polyposis with mepolizumab: randomized trial. Bachert et al. (2017) [16]	Mepolizumab (anti-IL-5)	*n* = 105 (Placebo *n* = 51 / Treatment *n* = 54)	CRSwNP	Asthma: *n* = 82	VAS, SNOT-22, adverse events, avoidance of surgery, endoscopic nasal polyp score, EQ-5D, Sniffin Sticks Screening-12, and lung function assessments.	- Significant improvement endoscopic nasal polyp score, all individual VAS symptom scores, and SNOT-22 score in the mepolizumab compared with placebo group - The was no statistically signification difference in olfaction via Sniffin Sticks Screening-12, and lung function tests -A reduction in blood eosinophil counts in the mepolizumab but not in the placebo
Efficacy and safety of dupilumab in patients with chronic rhinosinusitis with nasal polyps: results from the randomized phase 3 sinus-24 study. Han et al. (2019) [17]	Dupilumab (anti-IL-4/IL-13)	*n* = 276 (Placebo *n* = 133 / Treatment *n* = 143)	CRSwNP		VAS, SNOT-22, adverse events, patient-reported outcomes, ACQ-6, total nasal polyp score, UPSIT, FEV_1_, LMK Score, blood and serum markers	Dupilumab significantly improved nasal polyp score, LMK score, Snot-22 score, patient reported nasal congestion, and UPSIT scores from baseline compared to placebo. Asthma patients on dupilumab had improved lung function (FEV_1_) and ACQ-6 scores
A randomized phase 3 study, sinus-52, evaluating the efficacy and safety of dupilumab in patients with severe chronic rhinosinusitis with nasal polyps. Bachert et al. (2019) [18]	Dupilumab (anti-IL-4/IL-13)	*n* = 448 (Placebo *n* = 153 / Treatment Q2W/Q4W *n* = 145 Treatment Q2W *n* = 150)	CRSwNP		VAS, SNOT-22, adverse events, patient-reported outcomes, ACQ-6, total nasal polyp score, UPSIT, FEV_1_, LMK score, blood and serum markers	Dupilumab significantly improved nasal polyp score, LMK score, Snot-22 score, patient reported nasal congestion, and UPSIT scores from baseline compared to placebo. Asthma patients on dupilumab had improved lung function (FEV_1_) and ACQ-6 scores
Dupilumab reduces opacification across all sinuses and related symptoms in patients with CRSwNP Bachert et al. (2020) [19]	Dupilumab (anti-IL-4/IL-13)	*n* = 60 (Placebo *n* = 30 / Treatment *n* = 30)	CRSwNP	Asthma: Placebo *n* = 19 Treatment *n* = 16	zLMK, LMK Score, bilateral endoscopic nasal polyp score, UPSIT, SNOT-22, VAS, patient reported symptoms of nasal congestion and/or obstruction	After 16 weeks, Dupilumab significantly decreased opacification across all sinuses measured using the LMK and zLMK scoring systems, and significantly improved nasal polyp score, SNOT-22 score, VAS score, and UPSIT score At baseline opacification measured by total LMK score correlated with other assessed outcomes but not at 16 weeks

*CRSwNP* Chronic Rhinosinusitis with Nasal Polyposis, *CRSsNP* Chronic Rhinosinusitis without Nasal Polyposis, *NPIF* Nasal peak inspiratory flow, *Serum ECP* Serum Eosinophil cationic protein, *IL-5Rα* Interleukin- 5 receptor α, *IL* Interleukin, *MPO* myeloperoxidase, *SNOT-22* Sino-Nasal Outcome Test-22, *SF-36* 36-Item Short Form Survey, *UPSIT* The University of Pennsylvania Smell Identification Test, *RSOM-31* 31-item Rhinosinusitis Outcome Measure, *AQLQ* Asthma Quality of Life Questionnaire, *FEV*_*1*_ Forced Expiratory Volume, *PEF* Peak Expiratory Flow, *VAS* Visual Analogue Scale, *EQ-5D* Generic health-related quality of life questionnaire, *ACQ-6* 6-question Asthma Control Questionnaire, *ACQ-5* 5-question Asthma Control Questionnaire, *LMK* Lund-Mackay Score, *zLMK* Zinreich-modified Lund–Mackay Score

This white paper is meant to provide guidance in the use of biologic treatments for physicians trained in and experienced in providing both medical and surgical treatments for patients with CRS.

## Methods

The panel consisted of 17 fellowship trained rhinologists across Canada who are a part of the Canadian Rhinology Working Group within the Canadian Society of Otolaryngology-Head and Neck Surgery. Decisions were made from evidence-based recommendations but also reflected clinical expertise; therefore, a systematic literature search of all randomized control trials involving CRS and biologics were first obtained (Table 1) and disseminated to the group in order for the discussion and decisions to be driven by the evidence. The development of the recommendations were established through a Delphi model process.

This process involved three rounds of questionnaires in which the first two were completed individually online and the third was discussed on a virtual platform with all the panelists. The Round 1 questionnaire consisted of 28 provided statements that were established by AT, MD, IW, SK and are referred as the provided statements in the subsequent rounds. To remove bias, all participants were able to add new statements to fill in knowledge gaps that were not covered by the provided statements. The new statement additions were referred as panelist statements in the subsequent rounds. The 28 provided statements were evaluated on a scale of 1 to 10 in terms of a participant’s agreement with the statement. A rating within 1–3 indicated disagreement while a rating of 8–10 demonstrated agreement. A rating between 4 and 7 represented being neutral towards a statement. In addition to providing a rating, participants were encouraged to provide comments and add new statements as they deemed fit. All ratings were quantitively reviewed by mean, median, mode, range and standard deviation. Consensus was defined by removing the highest and lowest of the scores after both round 1 and 2 and using the “3 point relaxed system” [20]. This means, in order for a statement to reach consensus, all panelists’ ratings for the statement must fall within a 3-point region on the scale of 10. Statements that did not reach consensus were removed from the next round of statements but all results were shown to the panelists between each round.

## Results

After three rounds, a total of 11 statements achieved consensus. The statements that were removed throughout the Delphi process are not included here as this document only contains final agreed upon statements to provide the reader with clear statements regarding the use of biologics in patients with CRS. We expect this white paper to evolve over time and will require updating as additional clinical trials become available and clinical experience increases. Updated versions of this white paper will be available on www.entcanada.org.

## Discussion

### Consensus statements

1. *Patients must have both subjective and objective findings consistent with the diagnosis of CRSwNP to qualify for biologic therapy. All endotypes of CRSwNP are considered eligible except for primary ciliary dyskinesia and cystic fibrosis.*

Biologics have been largely studied in patients with CRSwNP. Therefore, patients who have been diagnosed with CRSwNP, based on the current Canadian clinical practice guidelines for CRSwNP, would be eligible [1]. CRSwNP is defined as having 2 or more of the following symptoms lasting at least 8 to 12 weeks [1]:

- Facial congestion/fullness
- Facial pain/pressure/fullness
- Nasal obstruction/blockage
- Purulent anterior/posterior nasal drainage
- Hyposmia/anosmia

Nasal endoscopy must show bilateral polyps within the nasal cavity to be considered for biologic therapy. Specialists must be cognisant that unilateral polyp disease can be caused by localized pathology such as fungal ball, antrochoanal polyps, odontogenic sinusitis or a tumor, both benign or malignant, and these diagnoses do not benefit from the use of biologic therapy [21]. For patients with primary ciliary dyskinesia or cystic fibrosis, these diseases are considered to have a disease pathophysiology that is not due to Type 2 inflammation; therefore, there is no perceived benefit in using currently available biologics that target Type 2 inflammation [21].

2. *Patients with CRSwNP do not need another Type 2 inflammatory condition such as asthma to be considered for biologic therapy.*

Historically, clinicians would provide biologics for patients suffering from asthma or atopic dermatitis and patients with CRSwNP indirectly benefited in this way. Both asthma and atopic dermatitis are Type 2 inflammatory diseases that have current indications for the use of biologics in Canada. However, currently there is clear evidence that patients with CRSwNP without asthma or atopic dermatitis benefit from biologic therapy. For instance, the efficacy of dupilumab was investigated in patients with CRSwNP regardless if they had any other Type 2 mediated diseases [15]. Dupilumab is a fully human monoclonal antibody to interleukin 4 receptor α inhibiting IL-4 and IL-13, which both play a central role in Type 2 inflammation. Individuals without asthma treated with dupilumab had a significant improvement in the secondary endpoints: total Sino-Nasal Outcome Test-22 (SNOT-22) scores, Lund-Mackay score, and objective olfactory scores compared to the placebo [15]. There was clinical improvement but no statistical significant improvement found in the primary endpoint of endoscopic nasal polyp score. However, those on dupilumab with comorbid asthma, having a more severe Type 2 disease, did have a significant improvement in nasal polyp score compared to the placebo [15]. These results are similar to those of the other three randomized control trials that included an asthma cohort but did not consider asthma as a criteria to participate [11, 13, 15].

3. *There is insufficient evidence to make a recommendation for providing biologics to patients with CRSsNP.*

There are no studies investigating the use of biologics in CRSsNP for the panel to consider. CRSsNP has not been studied, but the diversity of inflammatory profiles in CRSsNP suggests Type 2 inflammation may play a role in a subset of patients and trials are currently underway to assess the efficacy of this therapy.

4. *Biologics should not be provided to those suffering with recurrent acute bacterial sinusitis.*

There were no studies for the panel to consider that assesses the outcomes of biologics in the setting of recurrent acute bacterial sinusitis (RABRS) which is not considered a Type 2 inflammatory disease. Consequently, there is currently no recognized benefit for the use of biologic therapies in this patient group.

5. *The severity of subjective CRS symptoms needs to be moderate to severe based on the clinicians choosing of a validated patient reported outcome measure for chronic sinus disease.*

Examples of frequently used outcome measures for assessing subjective symptoms include, but are not limited to, the Chronic Sinusitis Survey (CSS), SNOT-22, Rhinosinusitis Disability Index (RSDI) and Visual Analogue Scale (VAS) [22]. In the eight randomized control trials that have been conducted on biologics targeting Type 2 inflammation in CRSwNP, most studies used the validated patient reported outcome, SNOT-22 [12, 15–18]. Otherwise a Visual Analogue Scale (VAS) score was used in combination or on its own [12–18]. Other studies used a “total symptom score” with a scale range of 0 to 9 points but this is not a validated outcome measure.

6. *Biologics should only be considered for those who have undergone adequate sinus surgery and failed appropriate medical therapy (AMT) following surgery. Patients unfit for surgery who have failed AMT may also be considered candidates for biologic therapy.*

Adequate sinus surgery that promotes ventilation, addresses mucostasis and facilitates topical medical therapy are essential goals in sinus surgery [21]. Following surgery, CRS patients should continue AMT. AMT is a term that has not been used in the current Canadian guidelines but in more recent guidelines from Europe have highlighted the importance of AMT [21]. The majority of CRSwNP patients will derive prolonged benefit from adequate surgery and AMT. As such, the panel recommends that ESS be used as an initial treatment modality in unoperated patients with CRSwNP who have failed AMT, as this provides a majority of patients with long-term disease control. However, patients who have already undergone ESS with minimal, or short-term improvement are at high risk of recurrence following subsequent surgery is performed. CRSwNP patients who have significant recurrence following ESS should be evaluated again to see if adequate surgery was performed and whether further surgery is required. Following this, they can then be considered for alternative therapies, such as biologics. Additionally, patients who cannot undergo surgery due to medical comorbidities but fail AMT may benefit from biologic therapies, as they cannot receive the full benefits of topical medical therapy due to unopened paranasal sinuses.

Surgery as a primary treatment modality remains cost-effective. In the United States it has been shown that for patients without a history of previous sinus surgery, ESS followed by AMT is more cost effective than primary dupilumab for CRSwNP patients [23]. This will likely be true for other biologics approved for CRSwNP given current suggested patient/drug plan costs. With the lower cost of routine outpatient sinus surgery in Canada (estimated to be approximately $3510.31 Canadian (CAD) per patient compared to an estimated annual cost for dupilumab of $25,918 CAD [24, 25]), this proposition continues to hold and informs this recommendation.

However, while fixing a dollar value for the first surgery and associated medical and work issues is relatively simple, it will be more difficult to fix a value for the repeated cycles of ESS and steroids accompanied by persistent symptoms these patients undergo otherwise. Thus, while the short-term costs of biologics exceed the costs of ESS for unoperated patients, for refractory patients with multiple recurrences the long-term value still remains to be determined.

A question emerges as to whether certain groups of patients may have demographic features or biochemical markers that could help identify those most likely to fail surgery in order to facilitate case selection for biologic therapy. Whether the biologic is best provided only after surgery despite these predictive features and/or marker or pre-emptively administered without surgery is unknown at this time. “Treatable traits” such as aspirin exacerbated respiratory disease (AERD) or markers such as peripheral eosinophils have been discussed but further research in this area is clearly still required.

7. *Option: A CT sinus scan performed prior to administration of biologics may determine if adequate sinus surgery was performed and to objectively confirm global mucosal inflammation.*

In patients unfamiliar to the clinician or in cases of uncertainty, a CT scan can help determine if adequate surgery was performed, or to rule out complications leading to diagnoses such as mucocele. Moreover, if on endoscopy it is unclear if the inflammation is isolated to a certain sinus or sinuses, a CT scan would help differentiate this. Biologics seem most beneficial for those patients with global mucosal inflammation. Inflammatory sinus disease of an isolated paranasal sinus may better benefit from corrective or extended surgical approaches to the site of interest for treatment.

8. *Response to biologics is based on subjective and objective improvement. Patients should experience an improvement to some or all of their major symptoms which include sense of smell, nasal obstruction, nasal discharge and facial pain. By 16 weeks, there should also be objective improvement on endoscopy or CT scan and this should be re-evaluated at 1 year.*

The definition of response is complex but requires subjective and objective improvement within a particular time frame. From completed trials, sixteen weeks appears to provide sufficient time to determine if the biologic therapy had a positive impact subjectively and objectively on a patient’s CRSwNP disease. The clinician may use the patient reported outcome measures used initially to define severity of symptoms to compare if there was a minimally important clinical difference in subjective symptoms. As for objective measures, the committee supports the use of endoscopy over CT scan. Clinicians are recommended to use a validated endoscopy grading rubric to help compare endoscopy findings before and after the 16 weeks of treatment. There is an appreciation for the limitation of polyp grading scales where there is a significant reduction in the size of the polyp and symptomatic improvement despite the polyp grade not improving with treatment, and this discordance needs to be considered in conjunction with the raw polyp grading score. There must be a discussion between the clinician and the patient to determine if the improvements achieved subjectively and objectively are worthy of continuing with biologic therapy.

9. *Providers have the option of providing another biologic therapy if patients fail to respond to one biologic agent but continue to fit the inclusion criteria for biologic therapy. At this time, there are no biological markers to determine the best biological agent to use.*

As of September 2020, there is only one biologic approved for use in Canada for CRSwNP and there are no studies that investigate outcomes following a switch in biologic therapy if a patient fails to improve with their first prescribed biologic agent. Since biologics target different inflammatory receptors and cytokines, patients may benefit from a biologic with a different target. The second biologic agent should be evaluated at 16 weeks for efficacy in the similar manner as the first biologic.

10. *Cost of biologics matters in the decision making of the use of biologics for CRS patients.*

In a single payer health care system, the cost of biologic therapy should be considered. Surgery remains a cost-effective option for most cases of CRSwNP. As the annual cost of biologics are high, their use should be restricted to appropriate cases where other options have been exhausted. Generally, biologics in Canada indicated for asthma can range between $600 to $4000 per vial/syringe, dependent on the drug [26, 27]. A cost utility analysis has shown that upfront surgery for CRSwNP is a more cost-effective option than dupilumab [23]. However, it is clearly evident that those who require revision surgery more than once will likely require it again and the time between surgeries diminishes with each surgery. Therefore, a cost utility analysis in this clinical scenario is required to address this question [6].

11. *The short-term use of biologics (12 months) in CRSwNP is considered safe. In other Type 2 inflammatory conditions, biologics have been shown to be safe long term.*

At this time, there is evidence from published studies that the use of biologics in CRSwNP are considered safe for short-term use up to 52 weeks. The most common adverse events reported include headache, nasopharyngitis, upper respiratory tract infection, oropharyngeal pain, and injection-site reactions [10]. No serious adverse events related to the use of biologics were reported [10]. However, the safety of biologics for other indications such as asthma and atopic dermatitis have been researched more widely and demonstrate that they are safe for long-term use over years of use, and millions of injections [28, 29].

## Conclusion

Management options for patients with CRSwNP now includes biologics. While biologics have been used for several years in other Type 2 conditions, they are novel in the management of CRSwNP. Discussion of biologic therapy for CRS treatment should be undertaken by a physician trained in and able to provide both medical and surgical treatments for CRS; who is able to undertake an informed discussion of the risks, benefits and possible complications of all of the treatment options with an affected patient. This white paper provides guidance to clinicians considering biologic therapies for CRSwNP, but the medical and surgical regimen should ultimately be individualized to the patient. As more biologics become available and additional trials are published, this white paper will be updated on www.entcanada.org with a revised published version every few years.

## Data Availability

The datasets used and/or analysed during the current study are available from the corresponding author on reasonable request.

## Abbreviations

CRS: Chronic rhinosinusitis
CRSwNP: Chronic rhinosinusitis with nasal polyposis
ESS: Endoscopic Sinus Surgery
IL: Interleukin
Ig: Immunoglobulin
CRSsNP: Chronic rhinosinusitis without nasal polyposis
AFRS: allergic fungal rhinosinusitis
SNOT-22: Sino-Nasal Outcome Test-22
RABS: Recurrent acute Bacterial Sinusitis
CSS: Chronic sinusitis survey
RSDI: rhinosinusitis disability index
VAS: Visual analogue scale
AMT: Appropriate medical therapy
CAD: Canadian
NPIF: Nasal peak inspiratory flow
Serum ECP: Serum eosinophil cationic protein
IL-5Rα: Interleukin- 5 receptor α
MPO: myeloperoxidase
SF-36: 36-Item Short Form Survey
UPSIT: The University of Pennsylvania Smell Identification Test
RSOM-31: 31-item Rhinosinusitis Outcome Measure
AQLQ: Asthma Quality of Life Questionnaire
FEV1: Forced Expiratory Volume
PEF: Peak Expiratory Flow
EQ-5D: Generic health-related quality of life questionnaire
ACQ-6: 6-question Asthma Control Questionnaire
ACQ-5: 5-question Asthma Control Questionnaire
LMK: Lund-Mackay Score
zLMK: Zinreich-modified Lund–Mackay
